# Phenotypic and Molecular Features of a Large ODDD Family: Expanding the Spectrum of CX43-Related Disorder

**DOI:** 10.3390/ijms27177655

**Published:** 2026-08-26

**Authors:** Irene Ambrosetti, Flavia Palombo, Diego D’Angeli, Danara Ormanbekova, Claudio Fiorini, Andrea Pietra, Carlotta Pia Cristalli, Raffaele Lodi, Caterina Tonon, Rocco Liguori, Valerio Carelli, Giovanni Rizzo, Alessandro Vaisfeld

**Affiliations:** 1 Clinical Genetics Unit, Azienda Ospedaliera Universitaria Integrata Verona, 37134 Verona, Italy; irene.ambrosetti@aovr.veneto.it; 2 Programma di Neurogenetica, IRCCS Istituto delle Scienze Neurologiche di Bologna, 40139 Bologna, Italy; flavia.palombo@ausl.bologna.it (F.P.); danara.ormanbekova@gmail.com (D.O.); claudio.fiorini@isnb.it (C.F.); valerio.carelli@unibo.it (V.C.); 3 Department of Biomedical and Neuromotor Sciences, University of Bologna, 40126 Bologna, Italy; diego.dangeli@studio.unibo.it (D.D.); andrea.pietra@studio.unibo.it (A.P.); raffaele.lodi@unibo.it (R.L.); caterina.tonon@unibo.it (C.T.); rocco.liguori@unibo.it (R.L.); 4 IRCCS Azienda Ospedaliero-Universitaria di Bologna, 40126 Bologna, Italy; carlottapia.cristalli@aosp.bo.it; 5 Functional and Molecular Neuroimaging Unit, IRCCS Istituto delle Scienze Neurologiche di Bologna, 40139 Bologna, Italy; 6 Clinica Neurologica, IRCCS Istituto delle Scienze Neurologiche di Bologna, 40139 Bologna, Italy; g.rizzo@unibo.it; 7 Department of Medical and Surgical Sciences (DIMEC), Alma Mater Studiorum, University of Bologna, 40126 Bologna, Italy; 8 Medical Genetics Unit, IRCCS Azienda Ospedaliero-Universitaria di Bologna, 40126 Bologna, Italy

**Keywords:** oculodentodigital dysplasia (ODDD), *GJA1*, connexin 43 (Cx43), hereditary paraplegia, hypomyelinating leukodystrophy, genotype-phenotype correlation, phenotypic expansion

## Abstract

We present a family of five siblings who came to our attention with a clinical and radiological diagnosis of familial hypomyelinating leukodystrophy. Despite brain white matter abnormalities being present in all siblings, the clinical phenotype was variable: the three brothers presented with a clear-cut late-onset spastic paraplegia, whereas the two sisters displayed only mild pyramidal signs. Molecular analysis revealed a single relevant variant shared by all affected siblings, namely the likely pathogenic variant c.659C>T (p.Ser220Phe) in the *GJA1* gene. Variants in this gene are generally associated with oculodentodigital dysplasia (ODDD), an autosomal dominant condition characterized by distinctive facial features and anomalies of the eyes, teeth, and digits. Neurological features are reported in about 30% of cases. In this family, ODDD manifested as a predominantly neurological phenotype. Although a clear explanation for this uncommon presentation is lacking, shared genetic modifiers, the effect of the specific variant, and a possible patient-population bias may have contributed. This case highlights the wide phenotypic spectrum of CX43-related disorders and suggests the importance of testing the *GJA1* gene in individuals with atypical presentations, including predominant or isolated neurological phenotypes such as late-onset spastic paraplegia. MRI findings may also provide a useful diagnostic clue when ODDD is suspected.

## 1. Introduction

Oculodentodigital dysplasia (ODDD) is a highly heterogeneous condition, classically characterized by a distinctive facial appearance, anomalies of the eyes (microphthalmos, microcornea), teeth (microdontia, agenesis, or premature loss of teeth), and fingers/toes (syndactyly, camptodactyly, brachydactyly) [1,2]. Other variably associated features include skin, hair and nail involvement (palmoplantar keratoderma, fine and sparse hair, brittle nails), cardiac findings (3% of patients), and lower-limb lymphedema [3,4,5,6].

Neurological involvement occurs in approximately 30% of patients, although the true prevalence may be higher due to underreporting or unrecognized mild symptoms [7,8].

Typical neurological phenotypes include spastic paraparesis/tetraparesis, cerebellar ataxia, dysarthria, neurogenic bladder dysfunction, epilepsy and mild intellectual disability. Brain MRI may reveal a wide spectrum of abnormalities, including hypomyelinating leukodystrophy, cerebellar/cerebral atrophy, thinning of the corpus callosum, and iron deposition or calcification [9,10]. Importantly, these MRI alterations can be present even in neurologically asymptomatic individuals and display a distinctive, disease-specific pattern that serves as a valuable diagnostic clue [11,12].

ODDD is caused by pathogenic variants in the *GJA1* gene, encoding connexin-43 (CX43), a ubiquitously expressed and functionally well-characterized member of the connexin family. This family comprises approximately 20 transmembrane proteins that assemble into hexameric hemichannels mediating intercellular communication through gap junctions, and when undocked, serve as conduits for cytosol-extracellular exchange [13].

Cx43 is highly expressed throughout the nervous system, where it sustains intercellular coupling and contributes to metabolic and ionic homeostasis [8,14,15]. Notably, Cx43 may contribute directly to CNS myelin maintenance by forming heterotypic gap junctions witholigodendrocyticCx47 (encoded by the *GJC2* gene) [16].

ODDD typically follows an autosomal dominant pattern of inheritance, although cases of autosomal recessive transmission (usually associated with a more severe phenotype) have been reported [17,18]. Given that the functional activity of the protein depends on the assembly of multiple subunits, the pathogenicity of *GJA1* variants in autosomal dominant ODDD is more likely explained by a dominant negative mechanism rather than haploinsufficiency [19,20].

In this paper, we describe a family of five siblings carrying a novel likely pathogenic variant in the *GJA1* gene, presenting with spastic paraparesis and leukoencephalopathy. In the absence of overt classical ODDD dysmorphic features, the diagnosis was only achieved through reverse phenotyping, following clinical exome sequencing in the proband and confirmation of variant co-segregation in the affected siblings.

## 2. Subjects and Methods

A family of five siblings (subjects 1–5) was evaluated clinically in the Neurology Unit of Bellaria Institute of Neurological Sciences in Bologna, Italy. All the siblings provided written informed consent for genetic testing, for the collection of photographs, and for publication.

Whole exome sequencing (WES) was performed on Subject 2 at IRCCS Istituto delle Scienze Neurologiche di Bologna for diagnostic purposes. The sample library was prepared from genomic DNA and enriched using xGenExomeHybPanel v2 (IDT) and sequenced on aNovaSeq6000 instrument (Illumina, San Diego, CA, USA) with 150 bp paired-end reads. Bioinformatic analysis followed the current best practices workflow for germline variant discovery [21]. Specifically, reads were aligned to the reference genome hg38; variants were called and filtered by quality with GATKHaplotypeCaller (https://gatk.broadinstitute.org/hc/en-us/articles/360037225632-HaplotypeCaller, accessed on 17 June 2024) and Variant Quality Score Recalibration (https://gatk.broadinstitute.org/hc/en-us/articles/360035531612-Variant-Quality-Score-Recalibration-VQSR, accessed on 17 June 2024) and then were annotated using Ensembl Variant Effect Predictor (VEP) (https://jun2026.archive.ensembl.org/info/docs/tools/vep/index.html, accessed on 17 June 2024). A panel of genes associated with Inherited White Matter Diseases (https://panelapp.genomicsengland.co.uk, accessed on 22 January 2024) was analyzed in silico. The variant was then segregated in the other siblings using Sanger sequencing. Variants were classified following the American College for Medical Genetics (ACMG) guidelines [22] with the online platform Franklin (https://franklin.genoox.com/clinical-db/home, accessed on 15 July 2024) and Varsome Premium (https://varsome.com, accessed on 15 July 2024). Structural analysis was performed using the AlphaFold-predicted model of human GJA1 (UniProt P17302). The p.Ser220Phe substitution was introduced by in silico mutagenesis in UCSF ChimeraX (https://www.cgl.ucsf.edu/chimerax/, version 1.12, accessed on 29 July 2026) using the Dunbrack rotamer library (https://dunbrack.fccc.edu/lab/bbdep2010, accessed on 29 July 2026). Wild-type and mutant models were compared to investigate potential local structural differences associated with the amino acid substitution. Residue contacts and predicted hydrogen-bond interactions involving residue 220 and neighboring amino acids were evaluated in both models using ChimeraX. The potential impact of the variant on protein stability was additionally assessed using the DynaMut2 web server (https://biosig.lab.uq.edu.au/dynamut2/, accessed on 29 July 2026), which estimates mutation-induced changes in protein stability (ΔΔG) based on structural and dynamics-based parameters.

## 3. Results

### 3.1. Genetic Testing

The clinical features and the familial recurrence of neurological symptoms in the five siblings were highly suggestive of a geneticetiology. Therefore, extensive molecular analyses were performed independently in subjects 1 and 2.

Two NGS panels for “spinocerebellar disease” and “neuromuscular disease,” respectively, were performed in subject2, bothcomprisingbetween160 and 170 genes related to neurological phenotypes but not including *GJA1*. No pathogenic/likely pathogenic variants were found; a variant of unknown significance (VUS) was reported in the *KIF5A* gene (NM_004984.4): c.1082C>T (p.Ala361Val). This variant was not considered causative of the phenotype, taking into account the lack of co-segregation with the disease in the family (the variant was present in siblings 2 and 5 but absent in sibling 1).

Subject1had undergone genetic testing elsewhere due to gait disturbance, which began around the age of 60 and was preceded by mild neurological symptoms. Although the molecular analysis reports are unavailable, the results were reportedly non-diagnostic.

Singleton wholeexomesequencingwasthenperformedonsubject2.

The analysis identified the heterozygous variant NM_000165.5: c.659C>T; p.(Ser220Phe) in the *GJA1* gene (ENST00000282561.3); the variant was segregated in the family and was found to be present in all five siblings.

**Figure 1:**
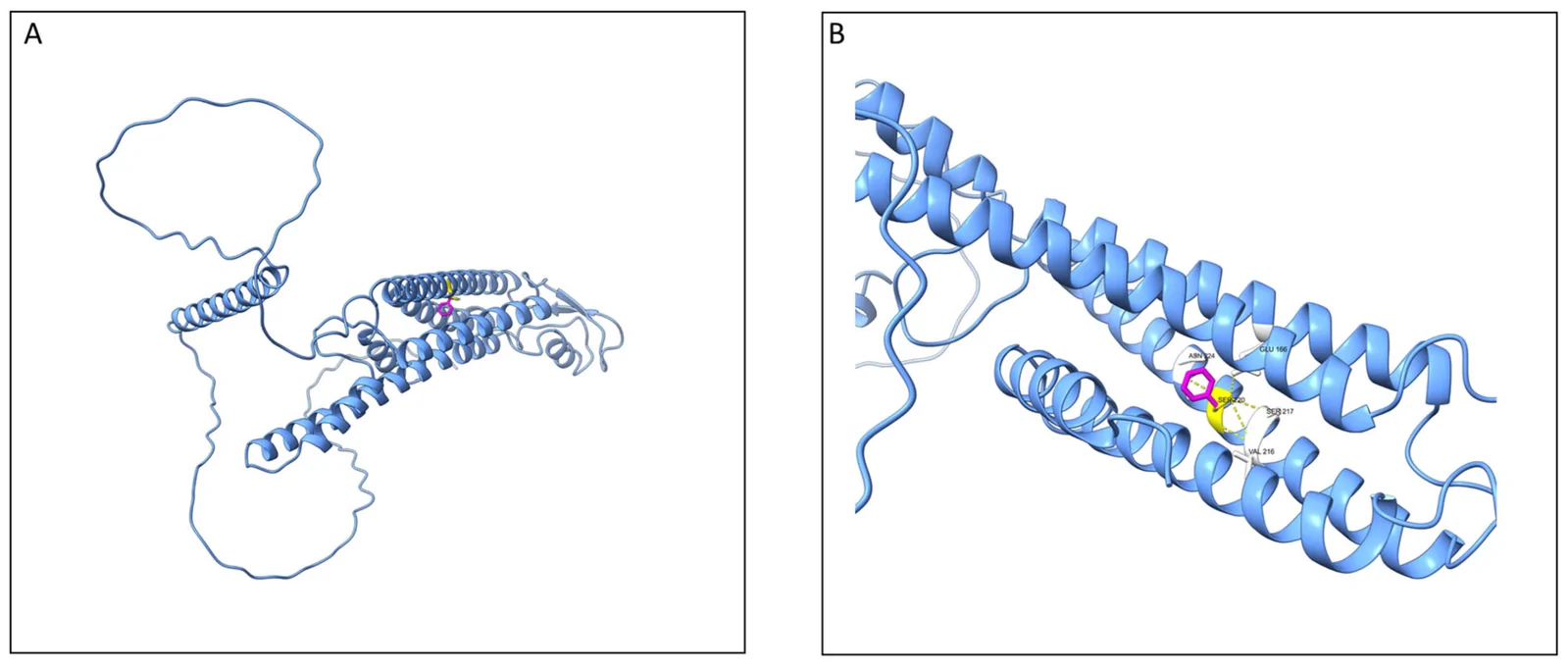
Structural modeling of GJA1 p.Ser220Phe. (**A**) AlphaFold predicted three-dimensional models of GJA1 structure showing the wild-type and mutant models. The position of the native Ser220 residue (yellow) and the substituted Phe220 residue (magenta) is highlighted, illustrating the location of the variant within the predicted protein structure. (**B**) Close-up view of the molecular environment surrounding residue 220. Ser220 (yellow) and Phe220 (magenta) are shown as sticks together with neighboring residues involved in predicted hydrogen-bond interactions.

This variant is classified as likely pathogenic according to ACMG criteria (PM1 moderate, PP2 supporting, PM2 moderate, PM5 supporting, PP3 supporting); it is absent in the latest version (v4) of the gnomAD database [23] (latest access on 14 March 2026 from https://registry.opendata.aws/broad-gnomad) and it was not reported in ClinVar nor, to our knowledge, had it been described in the scientific literature in association with ODDD. A different change affecting the same amino acid p.(Ser220Tyr) has been reported in an individual with ODDD [24]. In silico prediction analyses consistently supported a deleterious effect of the amino acid substitution. The variant was predicted to be pathogenic by multiple computational approaches, including AlphaMissense (score: 0.9912), REVEL (score: 0.931), ClinPred (score: 0.9968), EVE (score: 0.9895), and CADD (PHRED score: 25.6). Functional prediction algorithms such as SIFT (score: 0) also suggested a damaging effect. Structural analysis of the p.(Ser220Phe) variant showed that residue 220 is located within a high-confidence region of the predicted GJA1 structure (pLDDT = 91). The Ser-to-Phe substitution represents a non-conservative amino acid change, replacing a small polar residue with a bulky hydrophobic aromatic residue. Comparison of the wild-type and mutant models did not reveal evident major changes in the overall predicted protein architecture; however, inspection of the local environment surrounding residue 220 suggested a remodeling of the interaction network. In the wild-type model, Ser220 participated in five predicted hydrogen-bond interactions, including side-chain-mediated contacts involving Glu166, Val216, and Ser217. Following substitution with phenylalanine, the loss of the hydroxyl group resulted in the predicted disruption of these polar interactions, while backbone-mediated contacts involving Val216 and Asn224 were retained, resulting in a reduced number of predicted hydrogen bonds (5 versus 2) (Figure 1). Consistently, DynaMut2 predicted a mild destabilizing effect of the p.Ser220Phe substitution, with an estimated ΔΔG value of −0.46 kcal/mol. These computational findings suggest that the variant may affect the local chemical environment and interaction pattern surrounding residue 220, although the functional consequences require experimental validation.

### 3.2. Clinical Features

**Figure 2:**
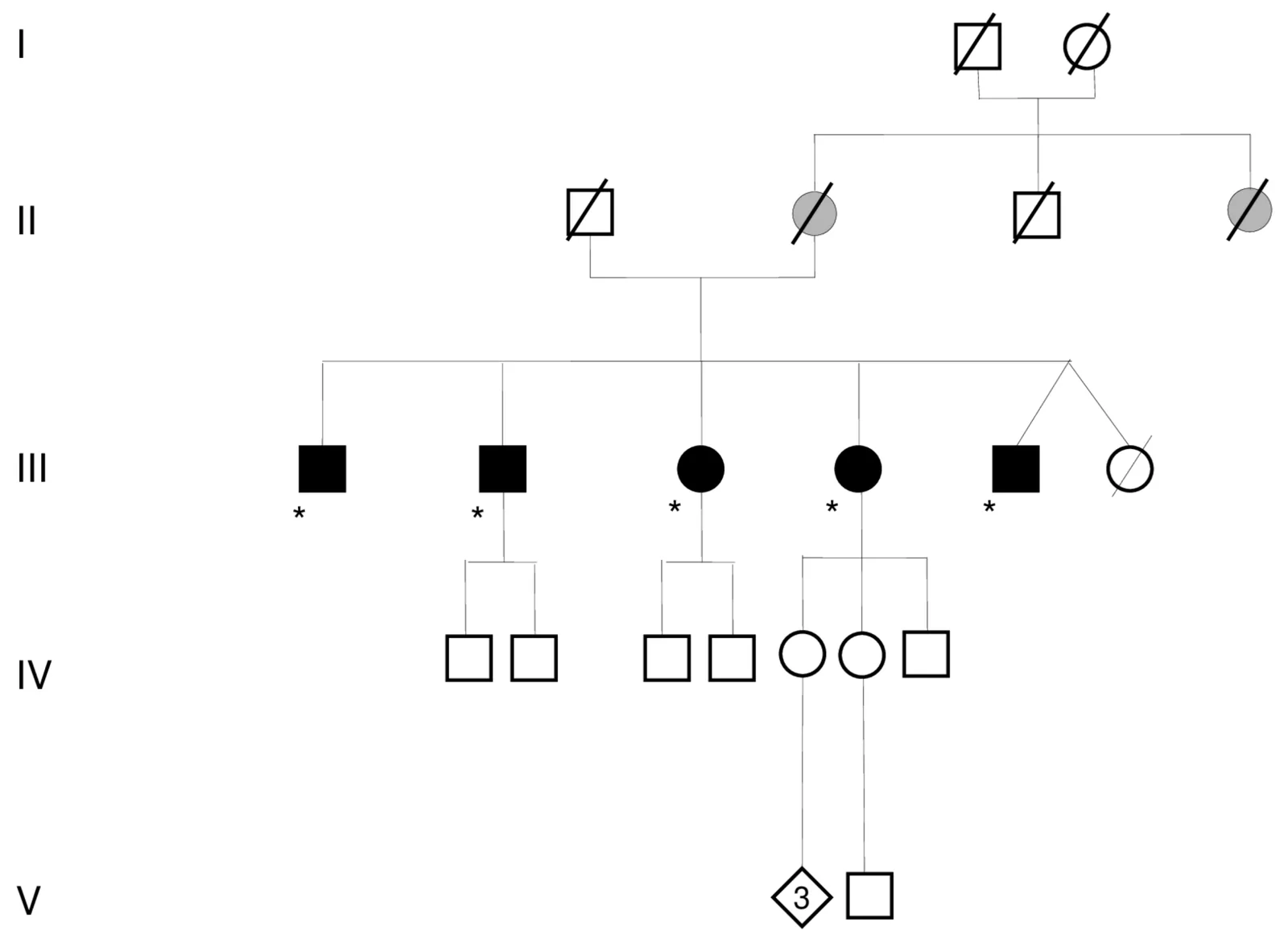
Pedigree. The siblings reported that their late mother (individual II2) showed gait disturbances with rigidity of the right leg, with onset before the age of 60. A maternal aunt (individual II4) also showed difficulty walking. * = *GJA1*:c.659C>T; p.(Ser220Phe).

Table 1 summarizes the clinical features of the siblings; neurological involvement was observed in all subjects, ranging from MRI-detected abnormalities in an otherwise asymptomatic individual (subject 4) to clinically overt spastic paraparesis in subjects 1, 2, and 5. Classical hallmark features of ODDD were not observed in the family; only subtle findings within the ODDD dysmorphic spectrum were identified retrospectively, after reaching a molecular diagnosis (Figure 2). A more detailed clinical description of each individual and their photographs are available upon request.

**Figure 3:**
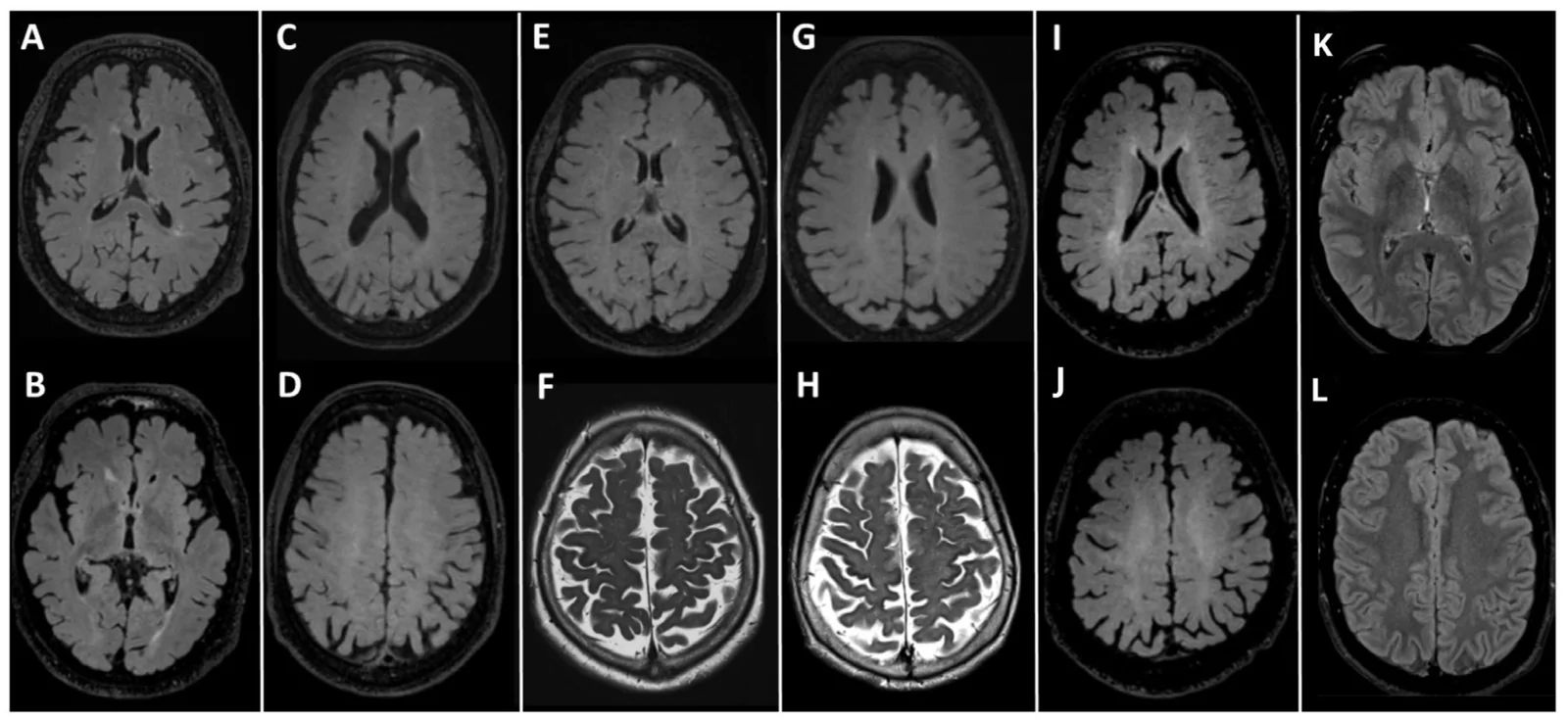
Axial FLAIR images demonstrate uniform white matter signal abnormalities with loss of normal gray-white matter contrast, consistent with hypomyelinating leukodystrophy in all subjects (**A**,**B**) subject 1; (**C**,**D**) subject 2; (**E**) subject 3; (**G**) subject 4; (**I**,**J**) subject 5; Axial FLAIR images (**A**,**B**,**I**,**J**) also show more pronounced symmetrical white matter hyperintensities with occipital predominance in two subjects; Axial T2-weighted images (**F**,**H**) demonstrate bilateral hypointensity in the Rolandic cortex in two subjects; note the normal gray-white matter contrast in FLAIR images (**K**,**L**) from a healthy subject, not carrying the variant, from the same family (son of subject 3).

Brain MRI was performed in all siblings (Figure 3). All individuals, including the asymptomatic sister, exhibited uniform white matter signal abnormalities with loss of normal gray-white matter contrast, consistent with hypomyelinating leukodystrophy. In two subjects, more pronounced symmetrical T2-hyperintensities were observed in the supratentorial white matter, particularly in the occipital regions. Additional findings included thinning of the corpus callosum and T2-hypointensitiesin the basal ganglia, dentate nuclei, and Rolandic cortex, suggestive of iron deposition.

## 4. Discussion

Clinical recognition and genetic testing for ODDD are typically prompted by the presence of hallmark dysmorphic features, such as syndactyly and microphthalmia. Here, we present a family in which the clinical presentation was predominantly neurological and therefore was not initially recognized as part of the ODDD phenotypic spectrum. Previous reports have documented families in which all affected members display a neurological phenotype, as well as individuals lacking the cardinal features of ODDD syndrome [7,10,18,25].

The family we have described presents a purely neurological clinical picture characterized by spastic paraparesis, albeit with a certain variability including an asymptomatic subject. Notably, all five siblings, including the asymptomatic sister, presented peculiar MRI changes consistent with hypomyelinating leukodystrophy.

The other findings consistent with ODDD, although supportive of the pathogenicity of the novel *GJA1* variant, were only recognized retrospectively following genetic diagnosis, with a reverse phenotyping approach and, in isolation, would have been insufficient to prompt genetic investigation.

It is possible that additional genetic factors such as modifier genes may contribute to the predominantly neurological phenotype observed in this family. Alternatively, we can hypothesize that the specific *GJA1* variant identified may exert a more deleterious effect on the nervous system; however, the available data are insufficient to support a genotype-phenotype correlation. This variant is located within the fourth (out of four total) transmembrane domain of the Cx43 protein, a region where other pathogenic variants have been reported, including a different missense change affecting the same amino acid residue, in association with the classical ODDD phenotype [24].

The in silico structural analysis performed in this study provides additional mechanistic support for the potential impact of the p.(Ser220Phe) variant on Cx43 structure. The substitution of the conserved polar Ser220 residue with a bulky hydrophobic aromatic amino acid results in the loss of the hydroxyl group and the predicted disruption of local polar interactions involving neighboring residues, including Glu166, Val216, and Ser217. Although the overall predicted protein architecture appears preserved, the altered hydrogen-bond network surrounding residue 220 suggests a possible modification of the local chemical environment and residue interactions. The mild destabilizing effect predicted by DynaMut2 (ΔΔG = −0.46 kcal/mol) is consistent with a potential contribution of the amino acid substitution to altered local structural dynamics. However, these findings should be interpreted with caution, as computational structural predictions provide mechanistic hypotheses rather than direct evidence of functional impairment. Experimental studies will be required to determine whether the predicted changes affect Cx43 folding, trafficking, gap-junction assembly, or channel function.

It is also possible that the exclusively or predominantly neurological phenotype we described is not as uncommon as previously thought in individuals with *GJA1* pathogenic variants, and that more individuals will be diagnosed with a *GJA1*-related disorder as this gene begins to be included in panels for neurological conditions or analyzed with Whole Exome or Whole Genome Sequencing approaches. This is what occurred in the case of this family, in which the initial genetic investigations, targeted toward neurological disorders, yielded non-diagnostic results.

Moreover, even within the context of a predominantly neurological phenotype in all individuals, the marked phenotypic variability, both in terms of clinical features and age of onset, should be emphasized: subject2 started noticing motor impairment at 30 years old, while his sister (subject 4) was 68 years old at the time of evaluation and only showed white brain matter abnormalities in the absence of neurological signs or symptoms.

One individual (sibling 1) was evaluated for infertility, as he and his wife were unable to conceive; he underwent surgical correction of varicocele and reportedly had oligospermia. Another brother (sibling 2) also reported fertility issues, although he was eventually able to father children. In the *GJA1* mouse model, subfertility in males due to reduced number and motility of sperm was observed [26]; to our knowledge, this characteristic has not been reported in humans so far. Although there can be many possible reasons for infertility, genetic as well as environmental or unknown, male infertility/subfertility could be a possible feature of *GJA1*-related disorders.

In conclusion, our experience underscores the importance of including ODDD in the differential diagnosis of different neurological phenotypes, especially spastic paraparesis, even when it presents as an isolated feature. The inclusion of *GJA1* in panels and in silico panels for neuromuscular disorders is strongly recommended, as non-neurologic features may be absent, mild, or appear only at a later stage. Moreover, MRI findings represent a valuable diagnostic clue and should be evaluated when ODDD is suspected.

## Figures and Tables

**Table 1:** Demographic and clinical features of the five siblings.

	Subject 1	Subject 2	Subject 3	Subject 4	Subject 5
**Demographic data**					
Sex	M	M	F	F	M
Age at genetic diagnosis	73	71	64	61	58
**Ophthalmological**					
Microphthalmia/microcornea	-	-	-	-	-
Short/narrow palpebral fissures	-	-	-	-	-
Strabismus	-	-	Mild	-	-
Glaucoma	-	-	-	-	Y
Other	-	Ptosis of left eyelid	Retinal dystrophy	-	-
**Dental**					
Enamel hypoplasia	-	Y	-	Y	Y
Microdontia	Y	Y	Y	Y	Y
Hypodontia	-	-	-	-	-
Abnormally spaced teeth	-	-	Y	-	Y
Other	-	-	-	Abnormal shape	Abnormal shape
**Digital**					
Syndactyly	-	Mild II–III toes	-	-	-
Clinodactyly	-	-	-	-	-
Camptodactyly	-	-	-	-	-
Brachydactyly	F/T	T	F/T	-	-
Dystrophic toenails	Y	Y	Y	-	Y
Other	Bilateral wide valgus I toe	Bilateral wide valgus I toe	Bilateral wide valgus I toe	Bilateral wide valgus I toe	Bilateral wide valgus I toe
**Neurological**					
Pyramidal signs *	Y (60 y)	Y (45 y)	Y (50 y)	-	Y (30 y)
Spastic paraparesis	Y	Y	-	-	Y
Neurogenic bladder	Y	Y	Y	-	Y
White matter abnormalities	Y	Y	Y	Y	Y
Other	Childhood seizures	-	-	-	Fecal incontinence
**Miscellaneous**					
Lymphedema of lower limbs	Y	Y	Y	-	Y
Reduced fertility	Y	Y	-	-	-

* Self-reported age of onset of pyramidal signs is in parentheses.

## Data Availability

The original contributions presented in this study are included in the article. Further inquiries can be directed to the corresponding author.

## References

[1] Kumar V., Couser N.L., Pandya A. (2020). Oculodentodigital Dysplasia: A Case Report and Major Review of the Eye and Ocular Adnexa Features of 295 Reported Cases. Case Rep. Ophthalmol. Med..

[2] Hindu K.D., Umer F. (2023). Oculo-dento-digital dysplasia: A systematic analysis of published dental literature. BDJ Open.

[3] Gumus E. (2018). A rare symptom of a very rare disease: A case report of a oculodentodigital dysplasia with lymphedema. Clin. Dysmorphol..

[4] Brice G., Ostergaard P., Jeffery S., Gordon K., Mortimer P.S., Mansour S. (2013). A novel mutation in GJA1 causing oculodentodigital syndrome and primary lymphoedema in a three generation family. Clin. Genet..

[5] Taki T., Takeichi T., Sugiura K., Akiyama M. (2019). Oculodentodigital Dysplasia Diagnosed from Severe Hypotrichosis. Acta Derm.-Venereol..

[6] Kogame T., Dainichi T., Shimomura Y., Tanioka M., Kabashima K., Miyachi Y. (2014). Palmoplantar keratosis in oculodentodigital dysplasia with a GJA1 point mutation out of the C-terminal region of connexin 43. J. Dermatol..

[7] Loddenkemper T., Grote K., Evers S., Oelerich M., Stögbauer F. (2002). Neurological manifestations of the oculodentodigital dysplasia syndrome. J. Neurol..

[8] De Bock M., Kerrebrouck M., Wang N., Leybaert L. (2013). Neurological manifestations of oculodentodigital dysplasia: A Cx43 channelopathy of the central nervous system?. Front. Pharmacol..

[9] Furuta N., Ikeda M., Hirayanagi K., Fujita Y., Amanuma M., Okamoto K. (2012). A Novel GJA1 Mutation in Oculodentodigital Dysplasia with Progressive Spastic Paraplegia and Sensory Deficits. Intern. Med..

[10] Amador C., Mathews A.M., del Carmen Montoya M., Laughridge M.E., Everman D.B., Holden K.R. (2008). Expanding the Neurologic Phenotype of Oculodentodigital Dysplasia in a 4-Generation Hispanic Family. J. Child Neurol..

[11] Harting I., Karch S., Moog U., Seitz A., Pouwels P.J.W., Wolf N.I. (2019). Oculodentodigital Dysplasia: A Hypomyelinating Leukodystrophy with a Characteristic MRI Pattern of Brain Stem Involvement. Am. J. Neuroradiol..

[12] Saint-Val L., Courtin T., Charles P., Verny C., Catala M., Schiffmann R., Boespflug-Tanguy O., Mochel F. (2019). GJA1 Variants Cause Spastic Paraplegia Associated with Cerebral Hypomyelination. Am. J. Neuroradiol..

[13] Van Campenhout R., Cooreman A., Leroy K., Rusiecka O.M., Van Brantegem P., Annaert P., Muyldermans S., Devoogdt N., Cogliati B., Kwak B.R. (2020). Non-canonical roles of connexins. Prog. Biophys. Mol. Biol..

[14] Nagy J.I., Rash J.E. (2000). Connexins and gap junctions of astrocytes and oligodendrocytes in the CNS. Brain Res. Rev..

[15] Jourdeuil K., Taneyhill L.A. (2020). The gap junction protein connexin 43 controls multiple aspects of cranial neural crest cell development. J. Cell Sci..

[16] Baracaldo-Santamaría D., Corrales-Hernández M.G., Ortiz-Vergara M.C., Cormane-Alfaro V., Luque-Bernal R.-M., Calderon-Ospina C.-A., Cediel-Becerra J.-F. (2022). Connexins and Pannexins: Important Players in Neurodevelopment, Neurological Diseases, and Potential Therapeutics. Biomedicines.

[17] Taşdelen E., Durmaz C.D., Karabulut H.G. (2018). Autosomal Recessive Oculodentodigital Dysplasia: A Case Report and Review of the Literature. Cytogenet. Genome Res..

[18] Joss S.K., Ghazawy S., Tomkins S., Ahmed M., Bradbury J., Sheridan E. (2007). Variable expression of neurological phenotype in autosomal recessive oculodentodigital dysplasia of two sibs and review of the literature. Eur. J. Pediatr..

[19] Huang T., Shao Q., MacDonald A., Xin L., Lorentz R., Bai D., Laird D.W. (2013). Autosomal recessive GJA1 (Cx43) gene mutations cause oculodentodigital dysplasia by distinct mechanisms. J. Cell Sci..

[20] Laird D.W. (2014). Syndromic and non-syndromic disease-linked Cx43 mutations. FEBS Lett..

[21] Van der Auwera G.A., Carneiro M.O., Hartl C., Poplin R., del Angel G., Levy-Moonshine A., Jordan T., Shakir K., Roazen D., Thibault J. (2013). From FastQ data to high confidence variant calls: The Genome Analysis Toolkit best practices pipeline. Curr. Protoc. Bioinform..

[22] Richards S., Aziz N., Bale S., Bick D., Das S., Gastier-Foster J., Grody W.W., Hegde M., Lyon E., Spector E. (2015). Standards and guidelines for the interpretation of sequence variants: A joint consensus recommendation of the American College of Medical Genetics and Genomics and the Association for Molecular Pathology. Genet. Med..

[23] Chen S., Francioli L.C., Goodrich J.K., Collins R.L., Kanai M., Wang Q., Alföldi J., Watts N.A., Vittal C., Gauthier L.D. (2024). A genomic mutational constraint map using variation in 76,156 human genomes. Nature.

[24] Paznekas W.A., Karczeski B., Vermeer S., Lowry R.B., Delatycki M., Laurence F., Koivisto P.A., Van Maldergem L., Boyadjiev S.A., Bodurtha J.N. (2009). GJA1 mutations, variants, and connexin 43 dysfunction as it relates to the oculodentodigital dysplasia phenotype. Hum. Mutat..

[25] Lopriore P., Vista M., Maritato P., Ienco E.C., Bassani L., Natale G., Tessa A., Santorelli F.M., Orsucci D. (2024). Deep neurological phenotyping in oculo-dento-digital syndrome. Neurol. Sci..

[26] Gregory M., Kahiri C.N., Barr K.J., Smith C.E., Hermo L., Cyr D.G., Kidder G.M. (2011). Male reproductive system defects and subfertility in a mutant mouse model of oculodentodigital dysplasia. Int. J. Androl..

